# Marital instability and its predictors in a representative sample of Mashhadi citizens, Iran, 2014

**Published:** 2015

**Authors:** V Vakili, H Baseri, Z Abbasi Shaye, MM Bazzaz

**Affiliations:** * Department of Community Medicine, School of Medicine, Mashhad University of Medical Sciences, Mashhad, Iran

**Keywords:** marital instability, predictors, Iran

## Abstract

**Background:**High quality and supportive relationships is essential to develop healthy individuals in all aspects of life. This study deals with the marital instability frequency and its predisposing factors and predictors in a representative sample of Mashhad, Iran, in 2014.

**Methods:**In a cross-sectional study, a total number of 583 couples participated. A checklist and the marital instability index (MII) were divided into 2 sections: the first section (part A) focused on the marital instability of couples and the second section (part B) focused on attractions and obstacles in each family, completed via a face-to-face interview.

**Results:**247 (42.2%) participants were male and 338 (57.8%) were female. The median age of participants was 34 years with a maximum of 82. The mean score of the marital instability was 19.97 ± 7.29 and 13.96 ± 3.08 for attractions and obstacles. Age, education, suspicion regarding the partner, history of divorce, the way to get married (personal or by family), socioeconomic concordance with partner, expressing love to partner and partner expressing love, were identified as predictors of marital instability. Sex, addiction, history of divorce, socioeconomic fit, family interfering, violence behavior and love expression regarding the partner, were predictors of attractions and obstacles part.

**Conclusion:**The overall score obtained from the marital instability questionnaire in this study was fair and showed lower levels of marital instability and divorce among our participants. However, it was necessary to inform young couple about the underlying factors of marital instability. Findings could be promising for the policy makers to design specific interventions suited to target population.

## Introduction

The high quality and supportive relationships is essential to develop healthy individuals in all aspects of life. Researches frequently depict individuals, who are in positive relationships, have better mental and physical health and performance [**1**]. Marriage, as an important custom, is a personal, but not private relationship with enormous public effects. A successful marriage is both men’s and women’s best bet for living healthy and happy. It provides the optimal conditions for bearing and raising children as well [**2**]. The current body of researches consistently depicted married women and men are both more likely to live longer, be physically and mentally healthier, and happier. Recovery from illness is quicker and more successful among them. Generally, mind their health and avoiding risky behaviors more [**3**]. Marital instability reveals affective and cognitive states in the company, with related actions, which predicted to terminate a relationship [**4**]. It is known that divorce or death can contain dissolutions, separations, dissatisfactions and even misunderstandings as well [**1**,**3**,**5**,**6**]. Marital instability has increased markedly in western developed countries since the mid-1960s. Despite the lack of consistent data, it seems that the same pattern has existed in Iran recently [**1**,**4**,**7**,**8**]. Nevertheless, social research is far from having clarified all of the micro- and macro-level predictors related to the marital instability. With this in mind, we found out the marital instability frequency and its predisposing factors and predictors in a representative sample of Mashhadi citizens, in 2014.

## Materials and Methods

In this cross-sectional study, we surveyed a total number of 583 couples in Mashhad, Iran, in 2014. Mashhad is the second most populous city in Iran and is the capital of Razavi Khorasan Province. It is located in the north east of the country, close to the borders of Afghanistan and Turkmenistan. Its population was 2,772,287 at the 2011 population census. The city is however most well known and respected for housing the tomb of Imam Reza, the eighth Shia Imam. Purposive and convenience sampling methods were adopted in selecting participants for this study. Purposive in the sense that only married individuals were used, and convenience in the sense that married individuals in different places who had time and expressed their consent in responding to our questionnaires, were used.

The survey was done by using a checklist and the marital instability index (MII) [**9**]. The checklist included the socio-demographic characteristics and factors related to the marriage of the respondents. The MII questionnaire consisted of 18 questions with Likert scale (from one (never) to 5 (always) scores) that were divided into 2 sections: first section (part A) focused on the marital instability of couples (14 questions) and the second section (part B) focused on attractions and obstacles in each family (4 questions). The score of 70 for part A and 5 for part B indicated the highest level of instable marriage and the score of 14 for part A and 20 for part B suggested the lowest level of marital instability. We used the Persian version of the questionnaire, being valid and reliable before [**10**]. The demographic information, including age, sex, education level, job status, and history of smoking, as well as drug or alcohol abuse, and etc., were asked in the checklist. We referred to the public transport stations, public parking lots, car parks of shopping centers, banks, hospitals and universities all around the city for data collection. The parking of Imam Reza holy Shrine was also a place for sampling the collection procedure. A total number of 583 questionnaires were completed. 

The Ethics Committee of Mashhad University of Medical Sciences approved the study. The interviewers explained the objectives of research for the participants and the latter were assured about the privacy of their personal data, and after getting the consent, they filled the questionnaires.

SPSS 11.5 software (SPSS Inc., Chicago, Illinois, USA) was used for all the statistical analyses. The standard descriptive statistics were applied to describe the pattern of the data. Chi-square test was used to examine the significance of the association between categorical data. The normality of the data was checked with Kolmogorov–Smirnov test. ANOVA and Kruskal-Wallis tests were also applied in normal and non-normal distributions respectively. Linear regressions were used to predict the factors’ influence on marriage instability. All the tests were 2-tailed, and the probability values less than 0.05 (p<0.05) were considered significant.

## Results

There were 583 participants in our research. The data showed that 247 (42.2%) participants were male and 338 (57.8%) were female. The median age of participants was 34 years with a maximum of 82 and minimum of 17 years. All of the respondents were Muslim; 572 (98.5%) of them were Shia and 9 (1.5%) were Sunni. The marriage durations were (median: 10, min: 0.5, max: 63 years). The engagement durations were (median: 1, min: 0.5, max: 2.1 years) and the age differences were (median: 1, min: 0, max: 35 years) among our participants. 240 (99.2%) male participants and 329 (99.7%) female participants had a modern way of marriage (without the help and support of parents), 205 (83.7%) males, and 302 (90.1%) females accustomed with the partner through family (Iranian traditional way of marriage). The frequency distribution of the participant’s demographic characteristics separated by gender was fully indicated in **Table 1** and factors related to the respondent’s marriage were shown in **Table 2**.

**Table 1:** Socio-demographic characteristics of study participants

		Man (n: 247)	Woman (n=338)
Age (median (min-max))		36 (22-78)	32 (17-82)
Occupation (n (%))	Self employed	129 (52.4)	46 (13.6)
	Employee	94 (38.2)	117 (34.7)
	Jobless/ housekeeper	3 (12)	140941.50
	Student	20 (8.1)	34 (10.1)
Education (n (%))	Illiterate	2 (0.8)	5 (1.5)
	Non academic	119 (48.2)	137 (40.8)
	Academic	126 (51)	194 (57.7)
Residential place (n (%))	Urban	235 (96.3)	325 (97.3)
Income (n (%))	<400US$	101 (54.6)	137 (75.3)
	400-800US$	58 (31.4)	37 (20.3)
	>800US$	26 (14.1)	8 (4.4)
Property owner (n (%))		140 (57.6)	202 (60.8)
Personal history (n (%))		13 (5.3)	12 (3.6)
Number of children(median (min-max))		1.5 (0-8)	1 (0-8)
Smoking (n (%))		39 (16.4)	61 (18.3)
Smoking duration (median ± SD͌1)		14.83 ± 10.30	14.23 ± 11.28
Alcohol consumption (n (%))		18 (7.4)	22 (7)
Alcohol duration (median ± SD)		13.18 ± 12.04	7.62 ± 2.83
Addiction (n (%))		10 (4.1)	23 (6.9)
Addiction duration (median ± SD)		9.89 ± 6.51	15.58 ± 1.5
Prison history (n (%))		5 (2)	6 (1.8)
*1= standard deviation*			

**Table 2:** Characteristics related to marriage

	Man	Woman	P-Value
Age at marriage (median (min-max))	25 (14-57)	21 (10-45)	<0.001
Family relationship (n (%))	77 (31.4)	81 (24.2)	0.05
Infertility History (n (%))	4 (2)	12 (3.7)	0.5
Suspicion to partner (n (%))	17 (7)	32 (15.9)	0.002
Divorce history (n (%))	14 (5.8)	17 (5.2)	0.01
Divorce in parents (n (%))	25 (10.2)	30 (9.1)	0.05
Pre-marital relationship with partner (n (%))	45 (20.8)	77 (22.9)	<0.001
Refer to counselor before marriage (n (%))	38 (15.4)	58 (16.8)	0.67
Cultural-ideological concordance with the partner’s family (n (%))	201 (81.7)	257 (76.7)	0.15
Socio-economic concordance with the partner’s family (n (%))	205 (83.3)	254 (75.8)	0.03
Agreement of your family for marriage (n (%))	228 (93.1)	300 (89.3)	0.12
Agreement of family’s partner for marriage (n (%))	236 (95.9)	308 (91.9)	0.05
Family's and friends’ Interfering (n (%))	46 (18.8)	69 (20.7)	0.56
Financial dependence on their family (n (%))	37 (15)	55 (16.4)	0.66
Expressed love to partner (n (%))	204 (83.3)	276 (82.6)	0.84
Receive love expressions from partner (n (%))	213 (86.9)	263 (78.3)	0.007
Violence behavior against partner (n (%))	29 (11.8)	46 (13.7)	0.50

 The mean score of marital instability in part A was 19.97 ± 7.29 out of the maximum 57 and minimum 14 and was 13.96 ± 3.08 with a maximum of 20 and minimum of 4 in part B. The median score of each question for men and women was shown in **Table 3**. For the females A2 and A4 questions, the highest score was received. Our analysis showed that questions A2, A4, A6, A8, A10 and B3 had a statistically significant difference between men and women (p-value <0.05/ **Table 3**).

**Table 3:** Marital instability aspects according to gender

Part A	**Man** (median)	**Woman** (median)	p-value
Sometimes married people think they would enjoy living apart from their Spouse. How often do you feel this way? (A1)	2	1	<0.001
Even people who get along quite well with their spouse sometimes wonder whether their marriage is working out. Have you ever thought: marriage might be in trouble? (A2)	1	2	<0.001
Have your spouse ever thought: marriage might be in trouble? (A3)	1	1	0.58
Have you ever talked With family members, friends, clergy, counselors, or Social workers about problems in your marriage? (A4)	1	2	<0.001
Has your spouse talked with relatives, friends, or a Counselor about problems either of you were having with your marriage? (A5)	1	1	0.82
Has the thought of getting a divorce or separation crossed your mind? (A6)	1	2	<0.001
Has the thought of getting a divorce or separation crossed your Spouse’s mind? (A7)	1	1	0.97
Have you or your spouse ever seriously suggested the idea of divorce? (A8)	1	1	0.01
Did you discuss about division of property? (A9)	1	1	0.47
Have you talked about filing a petition to an attorney? (A10)	1	1	0.03
Have you or your spouse consulted an attorney about a divorce or separation? (A11)	1	1	0.14
Because of problems people are having with their marriage they sometimes leave home either for a short time or as a trial separation. Has this ever happened in your marriage? (A12)	1	1	0.35
Have you talked about filing a petition your spouse? (A13)	1	1	0.12
Have you or your spouse filed a divorce or separation Petition at court? (A14)	1	1	0.45
Part B			
How often do you eat your main meal with your spouse? (B1)	4	4	0.27
How often do you meet your friends with your spouse? (B2)	3	3	0.78
How often do you help your spouse doing home activities? (B3)	2	3	0.01
How often do you go to cinema, party, travel with your spouse? (B4)	3	3	0.90

 After the analysis, we found that there were significant differences between the mean score of part A and smoking, alcohol and drug abuse (smoking, alcohol consumption and drug abuse can lead to instability, p<0.001), but there were no statistical differences between the mean score of part B and smoking and alcohol consumption. However, we had a significant difference between drug abuse and the mean score of part B (p<0.001).

To predict the concerning factors related to the marital instability, a linear regression by forward method was applied. Age, education, suspicion regarding the partner, history of divorce, the way to get married (personal or by family), socioeconomic concordance with the partner, expressing love to partner and partner expressing love, were identified as predictors of marital instability. Sex, addiction, history of divorce, socioeconomic fit, family interfering, violence behavior, and expressing love to partner were predictors of attractions and obstacles part (**Table 4**).

**Table 4:** Predictors of marital instability–linear regression

		Unstandardized Coefficients		Standardized Coefficients	t	p-value
		B	Std. Error	Beta		
Marital instability*	(Constant)	34.36	2.56		13.39	<0.001
	Family interfering	3.64	1.041	0.19	3.50	0.001
	suspicion	3.80	1.41	0.15	2.70	0.01
	divorce	8.01	1.75	0.24	4.57	<0.001
	Traditional way of marriage	-4.23	1.26	-0.18	-3.35	0.001
	Socioeconomic concordance	-2.91	1.07	-0.15	-2.71	0.01
	Express love to partner	-3.39	1.19	-0.15	-2.85	0.01
	age	-0.12	0.03	-0.20	-3.53	<0.001
	Expressing love to partner	-4.51	1.24	-0.21	-3.64	<0.001
	education	1.97	0.87	0.12	2.27	0.02
Attractions & obstacles**	(Constant)	11.65	0.72		16.20	<0.001
	Family interfering	-1.399	0.412	-0.196	-3.393	0.001
	divorce	-2.658	0.659	-0.225	-4.034	<0.001
	Socioeconomic concordance	1.352	0.404	0.184	3.344	0.001
	Expressing love to partner	0.96	0.45	0.12	2.161	0.03
	Violence behavior	-1.04	0.49	-0.118	-2.110	0.04
	Gender***	0.89	0.33	0.15	2.70	0.01
	addiction	-1.60	0.73	-0.13	-2.17	0.03
*=R2: 0.34						
**=R2: 0.23						
*** = reference: female						
*The negative states of variables were considered as references.*						

## Discussion

Research in recent years has shown that several factors may underlie the fragility of family bonds and the subsequent dissolution of the marriage, so the present study was conducted to investigate the instability of marriage and its underlying factors.

The study was conducted on 583 participants. The median age of marriage for men was 25 years and for women 21 years. The previous studies showed the marriage age as an affecting factor in the marital stability, which the age groups of 20-40 years were more susceptible to marital instability. One study depicted the highest divorce rate in the first 2-5 years of marriage [**11**], another study in Iran showed 43% of the reported conflicts were in the age range of 20-30 years. 57.4% of these couples were in the first 5 years of their marriage [**12**, **14**], this showed the importance of supporting and strengthening the modalities for the marital relationship in this sensitive period.

In the present study, the believing existence of trouble in marriage, talking or counseling with family members, friends, clergy, counselors or Social workers about problems in marriage, crossing the mind of the thought of getting a divorce or separation, seriously suggesting the idea of divorce with the partner, talking about filing a petition to an attorney and sharing the household chores, were significantly higher among women in comparison with men. Our findings showed that the overall women were more likely to have dissatisfaction from their marriage and were more likely to complain. It might be related to patriarchy. In a patriarchal society like Iran, power is primarily held by adult men. Males predominate in roles of political leadership, moral authority, social privilege and control of property; and, in the domain of the family, fathers or father figures hold authority over women and children [**15**]. Emotional differences between man and woman might be another related factor [**16**].

Religious beliefs and cultural adaptation are important factors in the stability of the family, as in the study of the causes of divorce, Rayhani and Ajam [**14**] reported that 73% of the marital instability predisposing factors are due to religious and cultural factors [**14**]. On the other hand, another study in Australia revealed higher levels of religious beliefs as a barrier for breakdown of marriage both among men and women [**4**], Yarnoz argued about the attachment of the human’s thoughts, perceptions and believes with his performance and interpersonal relationships [**17**]. Bottonari’s study admitted the relationship between the individual’s beliefs and marital instability as well [**18**]. However, in our study, no significant difference was observed.

We found in our study an economical in-concordance between wife, husband, and their families, as a predictor of marital instability. This finding was concordant with the previous studies. When there is economical concordance between couples, unrealistic expectations are less frequent. Usually, the point of view of themselves and their first degree family members are closer to each other, therefore, they do not prefer over another, and do not humiliate each other as well and generally the relationship is less challenging [**14**,**19**].

The parents’ interfering is another important factor that could cause trouble and fight among couples in our study. As Halford [**20**] showed, 10.6% of the divorce cases happened because of the families interfering [**20**]. In Iran, which is an Islamic country, the traditional form of marriage through families was more common previously. However, recent evolutions in technology, culture, economy and the educational level spatially among women as well as other environmental aspects had a deep influence of the way young men and women choose their spouse.

In present study, 11.8% men and 13.7% women reported spousal violence. Kulu [**11**] mentioned violence as the second-largest divorce predisposing factor in his study. Generally, men are more violent in spousal relationship and despite the compatibility with the wife; violence could lead to her dissatisfaction because she assesses her physical and psychological safety in danger. Violence arrows other adverse effects such as fear and anxiety for spouse and children as well, on the other hand, the acceptability of the couples among the family and neighbors have influenced [**11**]. We believe further studies warrant the attention in Iran.

Researchers have reported addiction, smoking, and drinking alcohol as the main causes leading to marital instability and divorce. On the other hand, Iran was faced with the rising trend of addiction rates in the world as well, the highest rate of heroin and opium addiction per capita worldwide reported from Iran: 1 in 17 is a regular drug user and 20% of Iranians aged 15 to 60 is involved in drug abuse [**21**]. In this study, the history of smoking, drinking alcohol and drug abuse had related with marital instability. The impact of these factors on the instability of marriage was expressed repeatedly previously. Halford’s study stated the addiction as 18.2 percent of the causes of divorce [**20**], Paul’s research also betrayed drinking alcohol, and using drugs, the most commonly cited reasons for the instability of marriage [**22**].

This study introduced the history of divorce, socioeconomic concordance among partners, family interfering, and education as predictors of marital instability. However, the strongest predictor was history of divorce. On the other hand, factors such as the traditional way of marriage thorough family, socioeconomic concordance with partner, love expression to partner were protecting factors. These results are similar to other studies [**13**,**23**,**24**]. In attractions and obstacles domain socioeconomic concordance, love expression to partner was shown by enhancing factors for marital stability, and family interfering, violent behavior, history of divorce, addiction was highlighted by reducing factors, and the strongest predictor was socioeconomic concordance between couples. These results are similar to other studies [**13**,**23**].

Given the results of this study, there is a growing need for planning about determinants of marital instability. Education regarding the criteria of spouse selection can be started very soon, even before marriage age, during school. Socioeconomic concordance among partners warrants consideration. Family interfering and love expression to partner are other important protective factors for marriage stability which are highly connected to education too. Based on our culture, we suggested young men and women, even in the modern way of marriage, to involve their family in their decision for marriage because having their support may prevent future interfering.

The strength point of our study was its generalization based on our best attempts to share a representative sample in this survey. However, we had restrictions in selecting a real representative sample; we suggested future studies to apply random sampling methods based on the total population to provide samples that are more representative. The average score of marital instability index, as well as details and predisposing factors mentioned clearly here, lack in other surveys from Iran.

This study had limitations, including the fact that people were reluctant to share their personal information. Sexual dissatisfaction played an important role in the instability of marriage, which was not possible to investigate in the present study. We suggested longitudinal design for a better understanding of this issue in future researches.

## Conclusion

The instability of marriage is one of the major problems in many developed and developing countries. The overall score obtained from the marital instability questionnaire in this study was fair and showed lower levels of marital instability among our participants. However, the determination of the underlying factors can help policy makers provide a better environment in order for couples to have a more stable relationship. Training workshops on life skills to raise the awareness regarding the factors contributing to the marital instability among young couples would be of interest.

**Conflict of interest**

The authors declared that there is no conflict of interest.

**Acknowledgements**

We kindly appreciate the efforts of all people involved in the project of recruiting participants and collecting the data. We thank all the participating couples for their cooperation and for providing personal information. This project was sponsored by Mashhad University of Medical Sciences
